# Copping out of novel feeds: HOW climate change pledgers and food summits overlooked insect protein

**DOI:** 10.1016/j.heliyon.2023.e22773

**Published:** 2023-11-24

**Authors:** Emmanuel Malematja, Nthabiseng Amenda Sebola, Tlou Grace Manyelo, Sekobane Daniel Kolobe, Monnye Mabelebele

**Affiliations:** University of South Africa, Department of Agriculture and Animal Health, College of Agriculture and Environmental Sciences, Florida, 1710, South Africa

**Keywords:** Affordable feeds, Animal feed commissioner, Climate change pledgers, Conventional feeds, Feed policies, Insect meal, Novel feeds, Sustainable diets, Unconventional feedstuff

## Abstract

The intention with this critical review is to appraise recent work done on insect proteins as animal feeds, and to discuss the possible factors which led to the ruling out of insect proteins by food and feed commissioners, as well as climate change pledgers. Of late, edible insect larvae have gained popularity as a promising protein source. On the basis of proximate analysis, insect species such as *Tenebrio molitor*, *Musca domestica*, *Acheta domestica*, *Zophobas morio* and *Hermetia illucens* have been reported to contain substantial amounts of protein, essential amino acids and minerals. Given these chemical properties, insects may be fruitfully utilized as a partial or sole protein source for monogastric rations. Although insect larvae hold immense potential as promising sustainable protein ingredients which are both ecologically and environmentally friendly, these unconventional feedstuffs are frequently overlooked and/or excluded from policies and legislation on feedstuff of animal origin, at local and international summits which pledge to develop sustainable food systems. Concerns about food insecurity, our expanding carbon footprint and deteriorating ecosystems, dictate that food and climate change summits bring to the mitigation table the concept of transitioning animal diets. A change must be effected from standard to sustainable diets, starting with a declaration on environmental impact and climate change concerns related to soybean cultivation and marine loss due to overfishing. The available literature on the chemical properties and environmental impact of fishmeal and soyabean meal production was scrutinized by accessing electronic databases and comparing these to insects’ nutritional composition and the impact which insect rearing has on the environment. As the literature search results revealed, information on the specific laws dealing with insect proteins as feed ingredients is scant, while the existing laws vary greatly. This has implications for innovation, as well as the trade in insect protein at a global level.

## 1 Introduction

Steadily rising economic growth, combined with rapid urbanization, has resulted in high demand for animal source foods or protein-rich food (Food and Agriculture Organization [FAO], [1]). An increase in demand for protein-rich food is also associated with biodiversity loss, land degradation and climate change [2]. Animal protein consumption has exerted intensified pressure on the global livestock production sector, with a corresponding aggravated negative impact on the health and wellbeing of humans and the planet [3,4]. Despite major strides being made in the efforts of researchers and technologists to promote the co-existence of natural resources and food production chains, there are worrying indications of biophysical unsustainability [5]. This is due to ongoing human population growth, climate change, a decrease in the number of wild-capture forage fish, and increasing competition between food and feed production entities [5]. Climate change is highly likely to affect global food production, and to have unpredictable consequences for food security [2]. These scenarios underline the need to search for alternative economical and sustainable feedstuffs, with a view to developing a sustainable food production chain [6,7]. Conventional protein production for use in animal feeds is being called into question, with a concomitant upsurge in the use of unconventional protein sources such as insect meal, which many view as the protein source of the future [8]. The FAO [1] has identified mass insect production as a potential source of feedstuff for animals. Insects are indeed an interesting food and feed ingredient to consider, for resource efficiency, sustainability and the circular economy, in the face of climate change [9]. Commercial interest in insects is spurred by their high nutritional content and vast potential use in diets [9]. Although insects are rich in nutrients such as proteins, lipids and minerals [10], their nutrient composition depends entirely on their species, their growth stage (larva, prepupa, pupa, nympha and imago), and their growth medium [11]. In addition to their high nutritive value, using insects in animal feeds has environmental benefits, such as converting large amounts of organic waste material into valuable nutrients [11]. Globally, various insect species have been investigated as protein sources in animal diets, and the findings are encouraging [12]. Notably, insect meal in poultry and fish diets is presumed to lower feed costs, thereby contributing to the cost-effectiveness of small-scale farming [2]. Other advantages of insects include their remarkably low carbon footprint, and the fact that their use will not result in resource exhaustion [3]. Insects have feasible breeding systems, and can be farmed throughout the year with limited resources [6,13]. Despite the enormous advantages of insects as an alternative and promising protein source, they are frequently overlooked at local and international summits hosted by animal feed commissioners, and at United Nations Climate Change (UNCC) summits which pledge to develop sustainable diets for humans and animals alike. The UNCC and the United Nations Food Systems Summit (UNFSS) conferences present an ideal opportunity to ensure that standard diets undergo sustainable shifts at the climate change mitigation table [14]. For that reason, any revised Nationally Determined Contribution (NDC) should include the concept of animal diet transitions – from standard to sustainable – starting with a declaration on the environmental impact of, and climate change concerns around, soybean cultivation and marine loss due to overfishing. That should ideally be followed by the identification of alternative feed ingredients, guided by the availability and nutritional composition of the proposed sources.

This study sought to determine the availability of conventional feed ingredients, the impact of soyabean and fishmeal production on the environment, global trends and concerns with regard to our expanding carbon footprint, and the availability of resources. Also discussed are the factors that may have prompted various stakeholders to rule out insect meals. Here, the focus also falls on the benefits of insect meals from an environmental and an animal nutrition perspective, and on how nations might meet the Paris Agreement's (– 2 °C above pre-industrial levels, but to no less than 1.5 °C) temperature goals. The overarching aim is to argue that developing sustainable livestock production chains will require a transition to unconventional feedstuff, such as mass-reared insects, in keeping with the principles and practices of livestock elevation.

## 2 Materials and methods

### 2.1 Electronic database access and search strategy

A systematic literature search was conducted by accessing data from electronic databases such as the Directory of Open Access Journals (DOAJ), Science Direct, Scopus, and Google Scholar. Since this review focused on the development of sustainable diets using insects to potentially substitute conventional feedstuffs in animal diets, in the face of climate change, the search process was extended to cover the role of animal feed commissions/summits in the search for sustainable protein sources. A literature search was conducted of recently published scientific articles in different journals. The databases were accessed using several keywords, each of which was paraphrased in different search engines. The keywords “alternative protein/s”, “conventional feed/s”, “insect meal/s”, “sustainable food production system/s”, “sustainable production chain/s” and “sustainable diet/s” were used. Citations included in articles from the databases were used to search for further relevant literature. In addition, inclusion and exclusion criteria were used in the second step of the process.

### 2.2 Article selection criteria (inclusion and exclusion criteria)

**Fig. 1:**
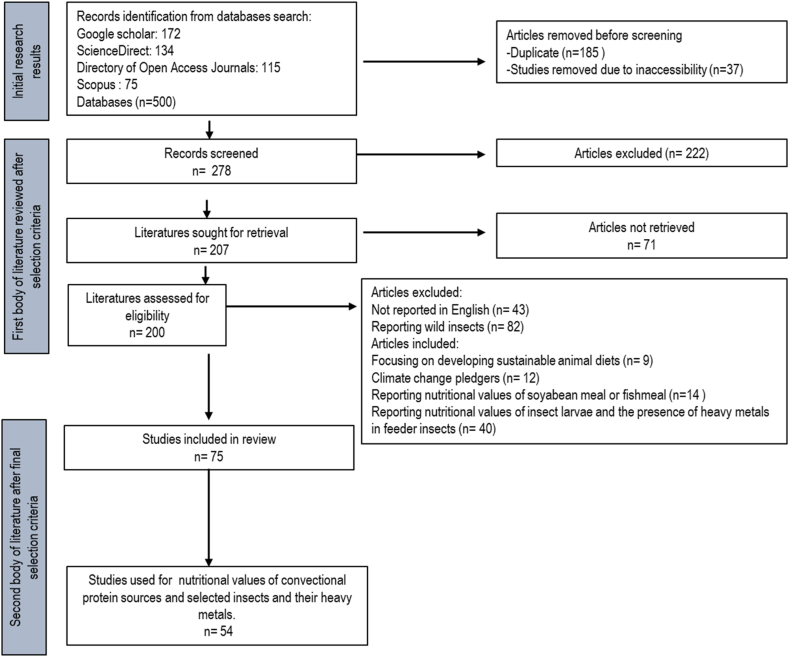
Prisma flow diagram summarizing the literature search strategy used in this systematic review.

The articles were selected following the criteria; including being consistently in English, available as open full-text, reported on the soyabean meal or fishmeal and insect meal as feed protein livestock. Furthermore, abstracts and full versions of these articles were read, evaluated and sorted using the inclusion criteria. Criteria for an article to be in included in the database were as follows; (i) the document is published in a peer-reviewed journal or book; (ii) articles that were recently published (from 2010 to 2023); (iii) articles reporting nutritional values of soyabean meal, fishmeal, and/or insect larvae. The final database consisted of 71 articles used in the whole study. Furthermore, the second selection criteria were employed which resulted in 50 articles that are specifically focused on selected 5 insect species and nutritional values as shown in Fig. 1.

## 3 Search results and discussion

### 3.1 Understanding the current state of global animal production trends: major components and driving forces

Recent findings indicate that global food production is being challenged by the ever-growing human population and by competition for high-quality agricultural land, with experts believing that the development of sustainable feed ingredients may present the fewest environmental risks and the highest efficiency in terms of non-renewable resources [15,16]. Addressing unsustainable food production is therefore a strategic necessity if the plan is to feed the global human population of about nine billion, while minimizing the impact on the environment and mitigating the effects of climate change [17]. Recently, animal production has emerged as one of the most important and fastest-growing agricultural sectors [5]. At the national level, livestock meat offers high-quality nutrients, significant opportunities for investment and numerous jobs, while also providing a source of income for smallholder farmers. The consumption of meat and meat products is projected to increase by 76 % by the middle of the 21st century, given the considerable shift in dietary preferences for protein-rich foods [17]. In addition, the global growing appetite for meat will require an expansion in livestock production, which will exert even more pressure on non-renewable resources [17]. The production of the majority of protein-rich foods (fish, meat, eggs and poultry) requires fishmeal and soyabean meal, hence the increased demand [18,19]. The demand for standard protein sources is expected to continue growing in the coming decades [1]. By contrast, the increase in global population growth, urbanisation, climate change, and the unsustainable use of input resources have exerted significant pressure on farmers, animal feed commissioners and governments around the globe, to transform the way animals are produced, distributed and consumed [20,21].

### **3.2** Aspects of resource management in terms of feedstuff availability and environmental impact

**Fig. 2:**
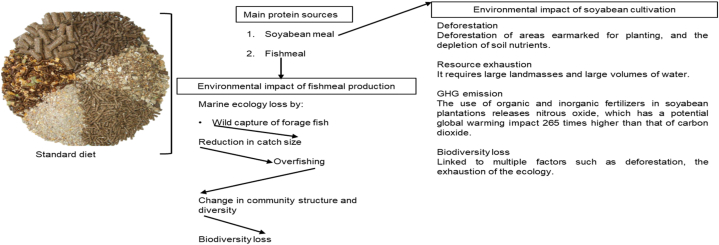
Environmental impact caused by soybean cultivation and fishmeal production.

Of late, the sustainable production of high-quality and sufficient protein has become a hot topic on global food security agendas. The production of protein-rich food is reliant on livestock production. Branches of the animal production sector – poultry, aquaculture and porcine industries – hinge on the use of highly digestible feedstuffs such as fishmeal, fish oil and soyabean meal [2]. Fishmeal in the diet represents a valuable protein source, with highly digestible amino acids to support growth in animals, while soyabean meal might supplements fishmeal in diets, but is required in large quantities [5]. The aforementioned conventional feed ingredient are the main sources of protein used in preparing monogastric rations, yet both are associated with erratic supply, fluctuating prices and, consequently, future shortages [16]. Soyabean (*Glycine max, L*), which is high in protein, is a plant-based protein source for use by both humans and animals, with 85 % of production destined for animal feed, and the remainder intended for human consumption worldwide [19,22]. The global soyabean production rate has doubled in the past two decades, with global production recorded at about 347 million tons in 2017/18 [18]. The top producer of soyabeans is the United States, followed by Brazil, Argentina and India [19]. Soyabean notably contains anti-nutritional components which negatively affect the nutritional quality of feed and prevents nutrient uptake and use, hence it cannot be fed raw to animals, without proper heat processing to improve its nutritional quality and the availability of nutrients [22]. The processing of soyabeans yields about 18.6 % of oil, and 78.7 % of soyabean meal [22]. Its acceptability in animal feeds is due to the high levels of crude protein content and almost equilibrate amino acid profile, all of which make the feed highly digestible and palatable [22]. In addition, soyabean is readily available throughout the year [19]. In soyabean meal, the amino acid composition is close to that of fishmeal, except in respect of methionine [22,23]. Importantly, as shown in Fig. 2 soyabean cultivation requires large landmasses and often the deforestation of virgin lands, the heavy application of fertilizers and pesticides, and large volumes of water [19]. From an economic and environmental point of view, the growing global demand for soyabean has a significant impact on soil fertility, causing soil erosion and desertification, as well as nutrient depletion which, in turn, results in biodiversity loss [19]. The surge in the use of soyabean meal as a major protein source in animal feed has thus been the driving force behind marked biodiversity loss, and is identified as a notable contributor to climate change.

From the onset of the twentieth century, fishmeal has been used as the main protein source in livestock production with very large quantity of fishmeal utilized as feed components in aquaculture and poultry feeds [24]. Fishmeal is trusted as the most valuable protein source in certain animal rations due to its high crude protein content [2], good amino acid profile, the fact that it is highly palatable and digestible, and its excellent nutritional composition in meeting the dietary requirements of both aquatic and monogastric animals – when compared to other protein sources [23]. Global annual fisheries production is recorded at 80 million metric tons, of which two-thirds are used for food, while the remainder is processed into fishmeal and fish oil [23]. Global fishmeal and fish oil production has been drastically declining since the start of the millennium, and by 2018 the global yield was 26.50 % lower than the fishmeal yield of 2000, which has led to an increase in both demand and price [23,24]. Most pelagic or forage fish species are overly sensitive to climate change, meaning their availability hinges on inter-annual, environmentally driven recruitment fluctuations [25]. Amongst the forage fish species used in fishmeal production are anchovy, capelin, sand-eel and sardines, whose numbers are currently declining due to overfishing and global warming [26]. Worryingly, the productivity of these species is declining at an alarming rate, with a 50 % reduction reported in the North Atlantic Ocean, and 20 % globally [23]. The aforementioned fish species play a significant role in animal feed manufacturing: the fishmeal and fish oil producers are therefore reliant on the availability and fast growth of forage fish, meaning the reduction in wild-caught fish has resulted in a shortage of the raw materials required to supply those industries.

### **3.3** Achieving food security in the face of climate change, adaption and mitigation

Currently, the main agenda in climate change and mitigation strategies at the local and international levels focuses on the development of sustainable food production practices, whereby food and feed can be produced in an environmentally efficient manner [6,27]. Importantly, the production of animal proteins requires a considerable quantity of natural resources, thereby contributing to climate change [28]. The Paris Agreement (COP 21) – a legally binding international treaty which entered into force in November 2016 – stemmed from a 2015 summit attended by numerous nations seeking to combat climate change, with almost all of them pledging to stabilize and mitigate global warming [29]. Their main target is to limit the global average temperature rise to – at most – 2 °C above pre-industrial levels, but to no less than 1.5 °C (United Nations Environment Program [UNEP], [30]). Worryingly, the current climate change prediction model indicates that the Paris Agreement commitments prefigure a 1.2 °C rise *above* the target agreed to by so many nations [21,31]. Therefore, for the goals of the Paris Agreement to remain attainable, strong commitments and rapid mitigations are needed. If left unabated, the main source of greenhouse gas (GHG) emissions in animal feed manufacturing will remain indirectly related to soyabean meal, fishmeal and fish oil, whose cultivation and production are also a major contributor to biodiversity loss [32]. The use of organic and inorganic fertilizers in soyabean plantations releases nitrous oxide, which has a potential global warming impact 265 times higher than that of carbon dioxide [28]. This begs the question: if the animal feeds and feedstuff commissioners or summit ratifiers endorsed and/or relaxed those laws and regulations which are imposed on novel feedstuffs, with a view to encourage their use in livestock diets, would this support the COP21 commitments, and close the gap between climate change mitigation and food security?

Today, the agricultural sector faces a range of complex challenges, including the decreasing availability of arable land and the depletion of water resources [33]. Meeting the Paris Agreement's temperature goals will require a gradual reduction in GHG emissions, and eventually, a need to decarbonize the animal feed industries [29,32]. Admittedly, it would be scientifically impossible for governments and food summit delegates to meet their goals under the Paris Agreement, to decarbonize the world economy (FAO and World Health Organization [WHO], [34]). Indeed, some governments have agreed to commit to the Paris Agreement's temperature-related goal of limiting global warming [32]. Significant consideration must, however, be given to the goals of both COP21 and global food summits, and how the use of novel feed ingredients may theoretically meet the world's food security needs, while keeping average temperature increases within the range committed to by the delegates to the Paris Conference. Climate change and the erratic supply of conventional feed ingredients have made insects the most appropriate substitute for soyabean meal and fishmeal [16]. The transition from conventional protein sources to novel proteins holds substantial potential for reducing production systems' environmental impact, and enhancing biodiversity through soil rehabilitation. From the point of view of the circular economy, introducing the use of insect meals in animal diets is an interesting topic for animal nutritionists, and the popularity of this option is expected to outstrip that of current conventional feed ingredients around the globe [33]. Using insect meal as feed ingredients may further help to solve socioeconomic and environmental problems such as land pollution, deforestation and marine degradation, in line with the United Nations' sustainable development goals [16]. Without proper intervention and action, soyabean meal production could become the main contributor of GHG emissions, driving levels above the goals set in the Paris Agreement. Overfishing is another concern, as it depletes the marine ecologies of oceans around the world [21,23].

### **3.4** Overview on literatures of nutritional values of commonly used insect larvae

FAO and European Union (EU) sources have articulated the potential use of insect larvae as alternative protein sources for animal feeds, and there are some indications that insects could provide a solution to the problem of securing a sustainable supply of feed ingredients [35]. Feeder insects commonly investigated as alternative protein sources for livestock include yellow mealworm (*Tenebrio molitor*), black soldier fly (*Hermetia illucens*), superworm (*Zophobas morio*), crickets (*Acheta domestica*) and housefly (*Musca domestica*). The larvae of these insects are relatively high in crude protein (40–60 %) and crude fat (10–20 %) [6], while their good amino acid composition is almost similar to that of conventional feed ingredients [36,37]. The most abundant indispensable amino acids in yellow mealworm include leucine, lysine, arginine and serine [38], with their nutritional composition reportedly comparable to that of fishmeal and soyabean meal [37]. These nutritional values are, however, not constant, and are determined by the insect species and the substrates in which they are reared, as well as the processing methods used [6,39]. For instance, Shumo et al. [40] observed that protein, fat, minerals and amino acids content of *Hermetia illucens* are influenced by organic substrate. The amino acid composition of *Zophobas morio* was influenced by the specific developmental stage [37]. Montowska et al. [35], having performed proximate analyses on cricket powder, noted that this insect species is rich in minerals such as calcium (Ca), magnesium (Mg) and iron (Fe). In similar vein, Mabelebele et al. [10] conducted a literature search study to review the availability of trace elements in edible insects, and observed that most contain sufficient amounts of calcium (Ca), phosphorous (P), iron (Fe), magnesium (Mg), zinc (Zn) and copper (Cu), to meet the mineral requirements of most livestock. Heidari-Parsa [38] also found a considerable amount of minerals and vitamins in both fresh and dried yellow mealworm. Ca, Fe and Zn are frequently inadequate in animal diets, hence insect larvae will arguably provide sufficient macro elements when included in livestock diets [41]. Insects accumulate most nutrients in their body at the larval stage, with crude fat being reported to be the second-most abundant nutrient, after protein [38]. Insects are also known to possess bioactive compounds such as chitin, silkrose and dipterose, which could be beneficial for the wellbeing and overall health of an animal through modulating intestinal microbiota [35,42]. Reported nutritional values of commonly utilized insect proteins are summarized in Table 1.

**Table 1:** Comparison between the nutritional composition of conventional protein sources and selected insect species (in percentage).

Composition	*Tenebrio molitor*	*Hermetia illucens*	*Musca domestica*	*Zophobas morio*	*Acheta domestica*	Soybean meal	Fishmeal
Crude protein	52.2–58.4	36.2–55.2	46.9–55.4	47.4–53.5	56.4–73.1	43.8–50.4	53–73
Crude fibre	6.3–11.4	7	6.2–7.1	3.0–7.5	3.9–7.6	4.3–7.2	0.8–1.5
Crude fat	29.4–32.0	10.2–22.9	20.8–31.3	38.0–40.0	15.9–15.3	1.0–3.0	8.2–9.2
Chitin	4.6–8.4	7.8–9.5	8.02	3.9	8.28–10.9	–	–
Ash	2.5–4.3	9.3–18.1	6.2–6.5	2.5–3.54	3.5–5.6	4.9–7.8	15.0–18.0
**Essential amino acids**							
Lysine	6.03	5.92–7.60	3.85–8.36	5.82	6.16–7.0	3.22–6.34	7.91–8.78
Methionine	0.64	1.50–1.70	3.00–4.03	0.76	1.49	0.5–1.01	2.93–3.02
Threonine	4.49	3.60–5.39	2.49–4.87	4.33	4.10–9.82	1.4–4.17	4.37–4.76
Tryptophan	4.18	6.35	0.69–5.79	6.28	4.91	0.51–2.93	1.18–3.91
Arginine	6.14	4.80–8.24	3.17–6.83	5.72	5.98–8.53	2.45–8.03	5.70–7.42
Isoleucine	5.87	4.16–5.76	1.84–4.89	6.36	3.69–5.31	1.76–5.47	4.74–5.04
Leucine	8.65	6.87–7.81	3.28–6.75	8.25	8.34–8.69	2.2–8.01	7.74–7.81
Valine	7.15	4.81–6.31	2.59–6.08	7.55	6.99	1.5–5.45	5.43–5.56
Histidine	3.64	2.71–5.29	1.59–4.68	3.87	2.34–2.93	1.0–3.28	2.41–7.86
Phenylalanine	4.29	3.60–6.88	3.82–7.01	5.0	3.28–4.23	1.6–5.79	4.12–5.38
**Author**	[15,43,44]	[24,45,46]	[[47], [48], [49]]	[44,50]	[43,51,52]	[[53], [54], [55]]	[53,55]

### 3.5 Traces of heavy metals in 5 selected insect larvae destined for feed protein

The key consideration with any newly introduced feed ingredient is the safety of, and acceptability by, animals, with such ingredients having to be free from contaminants and other undesirable substances such as toxins and heavy metals [6]. Bioaccumulation of undesirable substances and heavy metals in larvae body is obtained from feeding on contaminated substrates such as animal manure, and crop by-products [56]. These undesirable substances are of concern because they can easily enter human food chain and they have no purpose other than toxicity [57]. The presence of toxic heavy metals such as arsenic (As), cadmium (Cd), lead (Pb), and mercury (Hg) are often reported in insects' larvae [58]. The mentioned heavy metals are highly toxic, regardless of their lower dosages [59]. It has been demonstrated that the accumulation of these metals' hinges on insect species, growth stage, and the growing medium [56]. Edible insects such as *Tenebrio molitor, Hermetia illucens*, and *Zophobas morio* larvae have been reported to contain traces of Cd [60]. Purschke et al. [61] analyzed the larvae of *Hermetia illucens* for the presence of undesirable substances and reported lower concentration of Cd. This is in line with the findings by Elechi et al. [62] who also observed a low concentration of Cd. However, Bulak et al. [63] indicated that *Hermetia illucens* larvae is capable of accumulating high levels of Cd. Gao et al. [64] also reported small traces of Cd in *Musca domestica*. In another study it was reported that Pb concentration was below the detection level in *Tenebrio molitor* and *Zophobas morio* [60]. Nevertheless, these reported values met the European Union's concentration limit for Cd and Pb in feed ingredients. Furthermore, Charlton et al. [58] carried out analysis of As concentration in *Musca domestica* and *Hermetia illucens* and observed lower concentrations. In addition, some insects' species are known to possess Hg in their bodies [57], however, Hg can also be derived from substrates such as animal manure which are contaminated with vet drug residues. Traces of Hg have been reported in *Hermetia illucens* raised on various substrates [61]. Therefore, aforementioned concentrations met the current European Union (EU) and China maximum levels, thus indicating that insects' meals derived from yellow mealworm, black soldier fly, and superworm can be safely included in livestock feeds with less and/or without risk of heavy metal toxicity. *Acheta domestica* and *Zophobas morio* also contain chemical hazards, but less or few analyses have been done on this insect species. The present of heavy metal concentrations in insects' larvae are summarized in Table 2.

**Table 2:** Toxic levels and permitted limits of heavy metals in feed ingredients of animal-origin (mg/kg).

Heavy metal	*Tenebrio molitor*	*Hermetia illucens*	*Musca domestica*	*Zophobas morio*	*Acheta domestica*	General maximum limits	Maximum tolerable levels
Arsenic	0.021–0.023	0.024	0.079–0.191	–	0.02	2	≤2
Cadmium	0.0085–147	0.03–0.16	0.02–0.723	0.002–0.003	<0.005–0.02	2	<3
Mercury	0,0012–0.0049.1	0.012	0.002–0.038	–	–	0.1	≤0.1
Lead	0.063–0.079	0.55–2.68	–	0.002–0.004	0.09–0.25	10	≤10
**Author**	[60,62,65]	[24,61,62]	[58]	[41]	[66]	[67]	[68]

### **3.6** Influence of substrates on the nutrient composition of insect larvae destined for animal feed, and the environmental impact of insect production

**Fig. 3:**
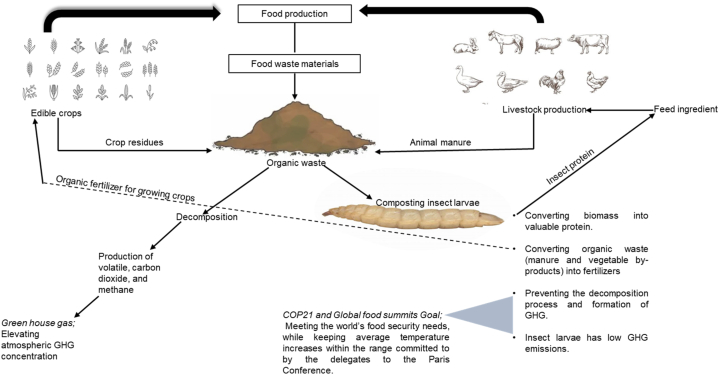
The impact which insect rearing has on the environment.

Volatile carbon dioxide and methane emissions are amongst the GHGs produced from decomposing animal manure, which contributes to environmental pollutants [13]. Decomposing manure is also associated with odour produced by butanoic acid, 3-methylbutanoic acid, skatole, organic sulphides, indoles, alcohol and volatile fatty acids [13]. These compounds, which are very harmful, pose potential health risks to humans [13]. In an attempt to alleviate this, various economic and environmentally friendly strategies have been considered [7]. As shown in Fig. 3, composting insect larvae has emerged as a greener path for waste treatment, with great potential to convert biowaste into valuable proteins for consumption [69]. Insect larvae can be reared on a range of low-cost substrates, including agricultural waste materials, animal manure and other biowaste materials [6,70].

Rearing insects on organic waste has proven to be a strategic way of preventing the decomposition process that will otherwise result in GHG emissions [6,13]. Pang et al. [7], who investigated the ability of insect larvae to reduce GHG formation from food wastes, observed that *Hermetia illucens* has the ability to prevent the formation of methane and nitrous oxide. Treating waste materials with *Hermetia illucens* larvae prevented the formation of ammonia gas, and reduced the total direct GHG emission from waste materials, when compared to the untreated group in that study [69]. The substrate chemical composition was, however, found to have a significant effect on performance and output [39], and, as demonstrated, various substrates yielded different results, regardless of the insect species [6]. The larval biomass obtained from composting insect larvae can be used in animal feeds as an alternative and sustainable protein source, to replace soyabean meal and fishmeal – two main, conventional feedstuffs proven to have detrimental effects on the environment [28]. This implies that it is possible to achieve the large-scale production of insect proteins for animal feeds, thus contributing to the circular economy by converting food and other organic waste (manure and vegetable by-products) into valuable outputs such as proteins and fat and fertilizers as additional strategy of the climate mitigation effect through insect rearing practices [6,71].

Due to their feasible breeding system, short life cycle, high output production rate, and low GHG emissions, insect larvae are considered to be an ecologically and environmentally alternative protein source for use in animal diets [37]. Given the aforementioned advantages, insect production or farming, when compared to the production of conventional protein sources, thus aligns with the goals of the Paris Agreement to mitigate the effects of climate change [11,16,29]. Despite the substantial economic and environmental benefits of using insect larvae, and the fact that the concept of insect farming for animal feeds has been intensively explored with promising results as a partial or complete replacement for soyabean meal in aquafeeds, porcine and poultry diets, a significant barrier to the uptake of insect meals remains.

### **3.7** Transition to unconventional feedstuff in the context of climate change, adaption and mitigation

Even with advanced technology and current research, there is a dearth of information on sustainable (human and animal) diets [72]. Sustainable food production, as defined by the World Commission on Environment and Development (WCED), involves the development of production systems that meet the needs of the current generation, without destroying or disturbing the ability of future generations to also attain their own needs [5]. There are many ways of developing sustainable production systems, including alternatives such as plant-based and cultured animal proteins [2]. For instance, integrating animal nutrition systems is an approach that will help to use and convert waste materials to valuable nutrients, and support both animal production and environmental conservation [71]. The question that arises, is how the use of insect meals as animal feedstuff would help to close the gaps between achieving food security, adapting to climate change and mitigating global warming, as natural resources such as water and arable land become increasingly scarce.

The proposed approach is aimed at adapting to climate change, while achieving food security. Food summits such as GIF Codex are working in conjunction with the FAO and the WHO to develop sustainable proteins. Their main goal is to develop a more sustainable, secure and equable protein supply, through the adaption of alternative or novel proteins such as plant-based, cultivated or fermentation-derived products, or cultured proteins of animal origin [34]. The aim of animal feed commissions is to ensure the safe use of feedstuffs of different origins in feeds, thereby bridging the gap between optimum livestock production and feed ingredient availability, to ensure the co-existence of natural resources, and food and feed production chains [34]. These commissions seek to interconnect the dimensions of sustainable agriculture, food security and climate change, adaption, and climate change mitigation. The wide availability, reasonable production cost and reliable nutritional content of insects have stimulated interest in these species as alternative protein sources [2]. Insects contain less antinutritional factors and more digestible fibre, which makes them an appropriate substitute for fishmeal in monogastric rations [6,70]. Recently, numerous methodologies have been inverted to increase the production of insects to a commercial scale. Commercializing mass-reared insects could help to develop a sustainable livestock production system, filling the gap between optimum productivity and production costs, mitigating famine and managing resources, reducing the impact on the environment and the carbon footprint, and offsetting the production and manufacture of conventional animal feeds [1,70]. Previously, a EU commission regulation (2021/1925) [73] authorized the commercialization of eight insect species, including black soldier fly, house cricket, common housefly, field cricket, yellow mealworm, bended cricket, silkworm, and lesser mealworm [11]. Vast tracts of literature speak to the positive effects of insect meals in aquafeeds and poultry diets, therefore insects also offer substantial potential for use in animal diets, and are now regarded as a promising feed source for future animal nutrition [70]. According to Wallace et al. [12], Selle et al. [18], and Selaledi et al. [2], insects such as yellow mealworm and other edible insect species offer an alternative protein source for poultry diets, without having a negative impact on growth performance and other parameters. Reportedly, replacing fishmeal with back soldier fly meal in diets positively affected the overall growth performance of guinea fowl [12]. For these reasons, animal feed commissioners should legislate the introduction of insect meal as a substitute for conventional protein sources, to formulate cost-effective diets. Moreover, there is a distinct possibility that insect meals will partially substitute standard protein sources in monogastric animals and reduce the reliability of animal protein production based on fishmeal and soyabean meal. If insect meals can partially replace standard protein sources in animal diets, animals’ nutrient requirements will be met with protein sources produced in an environmentally friendly manner.

### **3.8** Failure of animal feed summits and climate change pledgers to endorse the use of insect proteins in feeds

With the food production chain exerting tremendous pressure on natural resources, and scientific evidence identifying elevated levels of animal product consumption as detrimental to the environment and human health, the debate around alternative plant-based feed ingredients *versus* feed ingredients of animal origin has intensified over the past decades [72]. The argument made here, is that the debate should not revolve around plants and animal source feed ingredients, but food/feeds which contribute to sustainable diets [72]. Thus, the development of sustainable diets cannot be uncoupled from food production systems [1]. If their role in securing and ensuring the safe use of feed ingredients in animal feeds is so clear, why do stakeholders fail to acknowledge integrated feed system approaches, and the substantial potential use of unconventional feed ingredients?

Transformative and sustainable diets have come under discussion at a number of summits, including Conference of the Parties (COP21) in Paris, the work of the FAO and the WHO [34], Codex Alimentarius [21], Cop26 and 27 (From Climate Pledges to Transformative Action), United Nations Food System Summit (UNFSS), and various pledges and initiatives aimed at curbing climate change, preventing deforestation, ending ecological deterioration and promoting natural resource management. Notably, of the various pledges, Codex overlooked insects and their marked potential as an alternative protein source for both human and animals, when planning the development of alternative proteins during the annual review highlights conference in December 2021 [34]. Similarly, the joint summit consisting of the United Nations Food Systems Summit (UNFSS, 2021 conference), the United Nations Climate Change Conference (COP26), and the Nutrition for Growth Summit also overlooked insects as transformative feed ingredients in their pledges. The environmental impact of fishmeal and soyabean meal production will be reduced significantly if the use of insect meals in animal diets is legislated and presented at the various local and international summits aimed at shifting food production onto a greener path.

### **3.9** Developmental strategies for the animal feed and climate change summits, and recommendations

As indicated, current food and feed production systems threaten the environment, biodiversity, as well as the marine ecology [1]. From an economic and an environmental perspective, the “old way” of producing livestock is no longer sustainable, and the current production chain is uncertain. For that reason, it is vital to revisit the manner in which we farm animals, and this underlines the urgency to search for alternatives, and develop a sustainable production chain.

Given growing concerns about food insecurity, our expanding carbon footprint and deteriorating ecosystems, it is worth challenging the work of various commissions, by adopting a political viewpoint, an environmental perspective, and a view of sustainable animal nutrition. Climate change and the depletion of natural resources threaten future food production [1], and food insecurity is already affecting human wellbeing and economies around the world, and these problems are only projected to exacerbate in the coming decades [20]. With strategic changes, the animal nutrition sector can transform from advocating conventional or standard feed ingredients, to promoting integrated animal feeds, thereby mitigating climate change and transforming food systems [4]. To achieve food security, produce enough food for everyone and support the fast-growing livestock production sector, it is quite a challenging task to accomplish, without relying on standard feed proteins. However, the costs and unreliable availability of standard protein feedstuffs will increasingly prompt researchers to look for alternative, sustainable protein sources for use in animal diets. There are many opportunities for climate change adaptation in animal nutrition, including the development of sustainable animal diets [6], which reduce the environmental impact and minimize production costs. Importantly, the appropriate substitute protein source must possess good nutritional characteristics, including high levels of crude protein, low levels of fibre and few anti-nutritional components, while being highly digestible and palatable [23]. In addition, the selected alternative protein source must be widely available and affordable. Though various protein sources are available for use in animal diets, preference should be given to alternative, sustainable feed ingredients which are widely and locally available, to minimize transport costs [23]. This should exclude feed ingredients which are deemed to be food, to avoid competition between humans and animals [1,72]. Insofar as nutrient composition, environmental impact and climate change are concerned, the argument made here is that the legislation of insect meals in monogastric rations is vital. Introducing novel and sustainable feedstuff sovereignty constitutes a comprehensive approach aimed at transforming animal production systems and acknowledging the current role of sustainable animal production [2,72,74]. In addition to their highly nutritive values, insect meals present a strategic way of converting food waste biomass into valuable feedstuff, thus reducing methane formation. Therefore, when the environment, nutritional content and safe use of the proposed alternative protein sources are considered, there is no apparent contradiction. Introducing sustainable proteins such as insect larvae could promote the co-existence of natural resources and sustainable food production chains.

Animal feed summits have to stay relevant and keep up with the changing world of novel feeds – that includes insect meals and other plant-based proteins as alternative sources of protein. New animal feed and feedstuff policies are needed to reduce the environmental effects associated with their production, and to promote the shift towards sustainable animal diets.

### **3.10** Policies, existing laws and regulations on invertebrates as feedstuff of animal origin

As insects become increasingly popular as an alternative protein source for use in feed formulations worldwide, international guidelines on insect production and use in this regard should be as straightforward, fair and efficient as possible [34]. Although insect meals have remarkable potential as feed ingredients for use in animal feeds, specific laws are lacking, with existing legislation varying greatly [9]. Currently, there are no specific rules for feed and food hygiene, or microbiological criteria on insects as feed ingredients of animal origin [9]. Could the aforementioned factors be the reason why insect proteins have frequently been ruled out of the pledges of delegates at national and international summits, tasked with identifying sustainable food production systems?

It is thus worth looking at the rules which are applicable in different countries: in November 2022, the International Platform of Insects for Food and Feed (IPIFF) updated guide on good hygiene practices and approved the use of insect larvae reared on vegetable substrates for poultry, aquaculture and pigs in Europe [58]. Despite this, the rearing of insect larvae on animal waste or manure remains prohibited by current European law and regulations on the use of insects [6] – the EU currently only permits the use of insect larvae which are artificially reared on biomass [37]. While Australia has established the Insect Protein Association of Australia (IPAA) to appraise the use of insect protein in animal feeds, its regulations – set by Food Standards Australia–New Zealand (FSANZ) – require a newly introduced novel feed source to undergo pre-market assessment, and to be approved by FSANZ before being used or sold, unless previously approved. In Canada, there are no laws dealing with the use of insects in animal feeds, and insect-based diets for various pets are already available on the market [9]. China also permits the use of insect meal, and the existing regulatory framework for mass insect production promotes insect farming at scale [6]. By contrast, the use of insect feed in ruminant animals remains banned worldwide. It can thus be concluded that the specific laws on insect meals as feed ingredients are not sufficiently detailed and vary greatly within specific regions – two factors which have an impact on innovation and the trade in insects at a global level. In future, existing rules and regulations will have to be amended.

## 4 Limitations and perspectives with regard to the use of insect meal-laws and regulations

The use of animal-derived feed ingredients in animal feeds has the potential risk of transmitting spongiform encephalopathy and other prion diseases in animals, especially ruminants [6]. There are ongoing evaluations of feeder insects for use as protein sources in animal feeds [58]. Existing laws and regulations that ensure the safe use of animal feed ingredients by limiting undesirable substances in feedstuffs, are described in EC Directive 2002/32/EC [75]. This includes the presence of heavy metals, harmful compounds such as pesticides, veterinary drug residues, and other environmental contaminants [58]. With regard to the safe use of insect larvae in animal feeds, safety considerations hinge on the specific insect species, as there is an as-yet unknown risk of some insects containing natural metabolites that can be toxic to animals, when consumed [58]. However, the presence of veterinary drug residues in animal manure presents a major concern and holds a significant risk of proportion of insect larvae rearing for animal feed purposes, as demonstrated by prominent levels of heavy metals in the bodies of larvae [58]. For those reasons, the EU prohibits the use of animal manure as substrate in the production of insect larvae intended for feeding domesticated animals [6]. However, to date, no cases have been reported of insect meals carrying prions.

## 5 Conclusion

The transition to unconventional feedstuff is based on the enormous capacity to develop and implement sustainable solutions in animal production, which allow us to resolve several problems simultaneously, including uncertainties around conventional feed ingredients, high feed costs, resource management, and growing concerns about the expanding carbon footprint associated with food production. Soyabean meal and fishmeal are major conventional protein sources in monogastric rations due to their high crude protein content and good amino acid profile. Soyabean production and overfishing are, however, major causes of biodiversity destruction, and strongly contribute to climate change. In light of the limited natural resources available, and the threat of global warming, the production of major animal protein ingredients must become more sustainable, and ecologically and environmentally friendly. To provide sufficient high-quality feed ingredients and minimize the environmental impact of the production of conventional protein sources, several studies have identified the larvae of various insect species as alternative protein sources for livestock. Insect larvae are high in protein content (40–60 %), with adequate amino acids. Recently, they have been used with some frequency as a partial or complete replacement for conventional protein sources in animal feeds, with promising results. Nevertheless, this unconventional feedstuff is often overlooked and excluded from policies and legislation dealing with feedstuff of animal origin. Likewise, insects are frequently excluded from the agenda of local and international summits, where delegates pledge to develop sustainable animal diets and minimize the environmental footprint associated with food production. This creates a barrier for the uptake of insect proteins, along with the fact that no specific law has been promulgated on insect production and use.

## 6 Recommendations on the future direction of the insect industry

Given the dearth of information on specific laws governing insect meals as feed ingredients, and the lack of conformity in existing laws, current rules and regulations need to be revisited and amended, and global legislation has to be developed in respect of feeder insects, if we are to ease the global trade in, innovation regarding, and the uptake of, insect larvae for use in animal feed.

## Funding

National Research Foundation (grant number PMDS22052715122) and the 10.13039/501100008227University of South Africa for their financial support.

## Ethics approval

This study required no ethical approval.

## Data availability statement

No data were used for the research reflected in this article.

## CRediT authorship contribution statement

**Emmanuel Malematja:** Conceptualization, Investigation, Writing – original draft. **Nthabiseng Amenda Sebola:** Writing – review & editing. **Tlou Grace Manyelo:** Writing – review & editing. **Sekobane Daniel Kolobe:** Writing – review & editing. **Monnye Mabelebele:** Supervision, Writing – review & editing.

## Declaration of competing interest

The authors declare that they have no known competing financial interests or personal relationships that could have appeared to influence the work reported in this paper.
